# Systemic and Local Cytokine Profile following Spinal Cord Injury in Rats: A Multiplex Analysis

**DOI:** 10.3389/fneur.2017.00581

**Published:** 2017-10-31

**Authors:** Yana O. Mukhamedshina, Elvira R. Akhmetzyanova, Ekaterina V. Martynova, Svetlana F. Khaiboullina, Luisa R. Galieva, Albert A. Rizvanov

**Affiliations:** ^1^Institute of Fundamental Medicine and Biology, Kazan Federal University, Kazan, Russia; ^2^Kazan State Medical University, Kazan, Russia; ^3^University of Nevada, Reno, NV, United States

**Keywords:** spinal cord injury, acute and subacute periods, multiplex analysis, cytokine profile, rats

## Abstract

Our study of the changes in cytokine profile in blood serum and in the spinal cord after traumatic spinal cord injury (SCI) has shown that an inflammatory reaction and immunological response are not limited to the CNS, but widespread. This fact was confirmed by changes detected in a cytokine profile in blood serum samples [MIP-1α, interleukin 1 (IL-1) α, IL-2, IL-5, IL-1β, MCP-1, RANTES]. There were also changes in the levels of MIP-1α, IL-1α, IL-2, IL-5, IL-18, GM-colony-stimulating factor, IL-17α, IFN-γ, IL-10, IL-13, MCP-1, and GRO KC CINC-1 in samples of the rat injured spinal cord. The results underscore the complex cytokine network imbalance exhibited after SCI and show significant changes in the concentrations of 14 cytokines/chemokines with different inflammatory and immunological activities.

## Introduction

Currently, the treatment results of patients with traumatic spinal cord injury (SCI) are extremely poor. This field requires the development and implementation of new therapeutic protocols. Inflammation and tissue infiltration by various immune cells, which can penetrate into the spinal cord tissue through damage to the blood–brain barrier, play a significant role in the pathogenesis of secondary damage. At the site of inflammation there is an immunological dysfunction, of which the cause is still unclear. In addition, inflammatory processes lead to a systemic reaction and trigger an immune response that can potentially cause an autoimmune reaction similar to that of multiple sclerosis and central nervous system lesions in systemic lupus erythematosus. The question whether immune processes have a more positive or negative impact on regeneration is still a matter of discussion (1).

Thus, a search for modern methods to control degenerative processes in the central nervous system, based on the understanding of molecular mechanisms of pathogenesis of these disorders, and designed for a targeted delivery of immunomodulatory factors that are potentially capable of stimulating neuroregeneration is of immediate interest. The most complete evaluation of the immunological reactions after SCI affecting the course of posttraumatic processes has not been carried out, although it may potentially have good prognostic value and help a physician in choosing an optimal treatment strategy. Since inflammatory and autoimmune reactions aggravating processes of posttraumatic degeneration and inhibiting those of neuroregeneration lead to various negative results, it is extremely relevant to perform an expanded multiplex analysis of a cytokine profile to identify possible ways to modulate these posttraumatic reactions. Therefore, our research was aimed to study profiles of serum and spinal cord cytokines in an experimental animal model of dosed contusion SCI in acute and early periods after injury.

## Materials and Methods

### Animals

The study was specifically reviewed and approved by the Kazan (Volga Region) Federal University Animal Care and Use Committee guidelines (Permit Number 2, dated 5 May, 2015). All experimental procedures and protocols were consistent with the recommendations of the Physiological Section of the Russian National Committee on Bioethics. Rats were housed in clear plastic cages (12 h/12 h light/dark cycle) with food and water available *ad libitum*. In this study, we used 25 adult male Wistar rats (weight: 250–300 g each; Pushchino Laboratory, Pushchino, Russia) and assigned the animals randomly into experimental and control groups (Table 1).

**Table 1:** Experimental groups.

Groups of animals	Duration of experiment, days	Methods	Number of animals
Intact control	–	Multiplex analysis of blood serum and spinal cord at the Th8 vertebral level	5
Spinal cord injury	3	Multiplex analysis of blood serum and spinal cord in the area of injury	5
	7		5
	14		5

### SCI and Postsurgical Care

Rats were deeply anesthetized with an intraperitoneal injection of chloral hydrate (80 mg/ml, 0.4 ml per 100 g, Sigma). A dosed contusion SCI at the Th8 vertebral level was performed as described (2). After surgery, rats had a daily intramuscular administration of gentamicin (25 mg/kg, Omela, Russia) for seven consecutive days. Bladders of injured rats were manually emptied twice daily until spontaneous voiding occurred.

Venous blood (0.5–0.7 ml) was collected by catheterization of the caudal vein 2 h before the surgery (intact control) and then 3, 7, and 14 days after the SCI. After coagulation for 20 min, the blood was centrifuged at 3,000 rpm for 5 min. Blood serum was aseptically collected and frozen at −80°C until cytokine measurement. Animals with signs of surgical wound inflammation were excluded from the experiment.

To evaluate the spinal cord cytokine profile in intact animals as well as in animals with the SCI 3, 7, and 14 days after the injury, a portion of the spinal cord at the site of injury/Th8 5 mm in length was dissected and homogenized with an electric homogenizer adding 300 µl of a complete extraction buffer. The blade was rinsed twice with 300 µl of complete extraction buffer for each rinse, and constant agitation was maintained for 2 h at 4°C. After centrifugation for 20 min at 13,000 rpm and at 4°C, a soluble protein extract was collected and frozen at −80°C until the time of cytokine measurement.

### Cytokine Assay

Multiplex analysis based on the xMAP Luminex technology was performed with the use of a kit for Bio-Plex Pro™ Rat Cytokine 24-plex Assay # 171K1001M (Bio-Rad), according to the manufacturer’s instructions. Experiments were performed in triplicate. The kit enables a simultaneous multiplex analysis of 24 cytokines, chemokines, and interleukins rat in a 50-µl sample.

### Statistical Analysis

Data are presented as mean ± SEM. A one-way analysis of variance (ANOVA) with a Tukey’s test or a two-way ANOVA was used for multiple comparisons between all experimental groups. For the time course experiments, changes over time were assessed with one-way ANOVA followed by *post hoc* Bonferroni’s multiple comparison tests. A value of *P* < 0.05 or *P* < 0.01 was considered statistically significant. The data were analyzed with the use of Origin 7.0 SR0 Software (OriginLab, Northampton, MA, USA).

## Results

### Cytokine Profile in the Area of SCI

**Figure 1:**
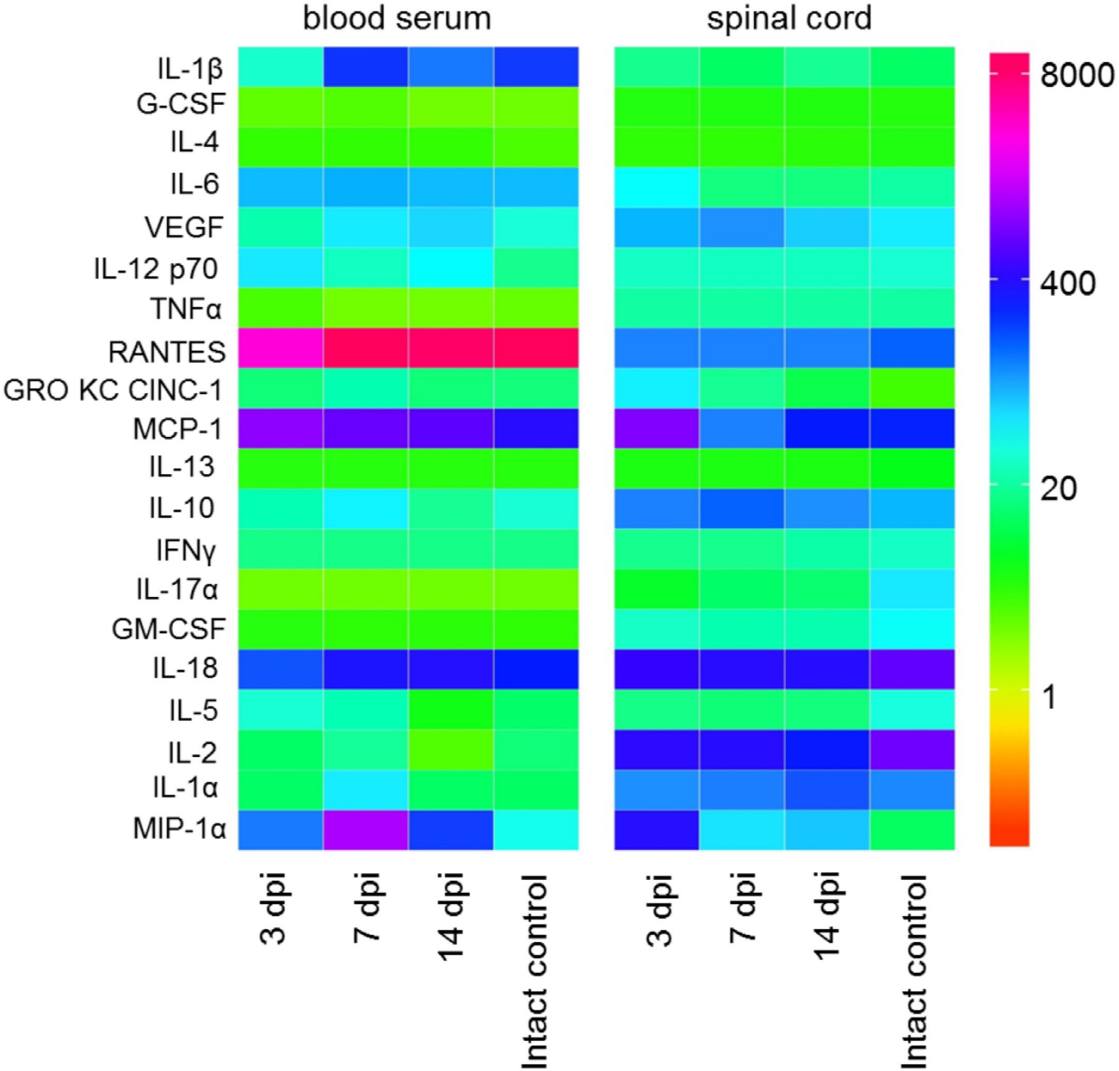
Heat map analysis of blood serum and spinal cord cytokine profile after spinal cord injury (SCI) in rats. Cytokine profile at days 3, 7, and 14 after SCI was analyzed.

The assessment of the cytokine profile in the site of SCI at different periods after contusion injury showed a significant change in concentrations of cytokines/chemokines such as MIP-1α, interleukin 1 (IL-1) α, IL-2, IL-5, IL-18, GM-colony-stimulating factor (CSF), IL-17α, IFN-γ, IL-10, IL-13, MCP-1, and GRO KC CINC-1 (Table 2; Figure 1).

**Table 2:** The spinal cord cytokine concentrations (pg/ml) at different stages after spinal cord injury.

	3 dpi	7 dpi	14 dpi	Intact control
MIP-1α	374.52 ± 103.96**	52.75 ± 30.95*	66.69 ± 40.23*	11.93 ± 6.91
Interleukin 1 (IL-1) α	104.4 ± 24.2	119.62 ± 32.8	174.53 ± 26.68*	110.08 ± 12.43
IL-2	407.52 ± 11.46*	380.21 ± 117.34*	273.48 ± 50.76*	750.87 ± 45.69
IL-5	16.55 ± 1.62*	14.3 ± 1.55*	15.1 ± 2.66*	33.63 ± 13.53
IL-18	471.09 ± 67.28*	391.68 ± 63.22*	371.3 ± 82.04*	659.22 ± 67.28
GM-colony-stimulating factor (CSF)	27.59 ± 3.11*	22.84 ± 3.83*	22.65 ± 1.75*	40.59 ± 3.4
IL-17α	7.6 ± 0.23*	12.76 ± 6.81*	13.73 ± 7.82*	50.65 ± 15.9
IFN-γ	17.32 ± 1.05*	17.07 ± 0.81*	21.64 ± 10.29	27.07 ± 4.86
IL-10	116.88 ± 30.97	148 ± 28*	103.58 ± 38.37	75.76 ± 25.47
IL-13	<5.65*	<5.65*	<5.65*	6.88 ± 0.31
MCP-1	902.7 ± 387.35*	117.03 ± 61.98*	271.17 ± 70.38	255.46 ± 23.64
GRO KC CINC-1	47.66 ± 10.05**	18.23 ± 9.54*	9.77 ± 5.12*	3.58 ± 0.62
RANTES	<115	<115	<115	149.29 ± 41.7
Tumor necrosis factor (TNF) α	<20.4	<20.4	<20.4	<20.4
IL-12 p70	27.32 ± 1.65	<26.5	<26.5	30.59 ± 3.41
VEGF	76.28 ± 25.09	101.78 ± 63.25	63.76 ± 12.97	49.04 ± 17.71
IL-6	41.75 ± 17.09	<15.44	<15.44	20.96 ± 9.56
IL-4	<4.22	<4.22	4.51 ± 0.59	5.33 ± 0.72
G-colony-stimulating factor (CSF)	5.17 ± 0.22	5.45 ± 0.21	5.22 ± 0.32	<5.06
IL-1β	17.28 ± 5.81	12.5 ± 0.7	18.13 ± 7.51	12.4 ± 3.1

*P < 0.05, **P < 0.01 as compared with the intact controls.

*<, protein concentrations below the specified value cannot be detected with the kit*.

On day 3 post injury (dpi), the MIP-1α concentration at the site of SCI dramatically increased more than 31 times when compared with intact controls (*P* < 0.01). On 7 and 14 dpi, there was a decrease in MIP-1α concentrations compared with the values on day 3; however, the values still exceeded those in the intact controls by 4.42 and 5.6 times, respectively (*P* < 0.05). There was a gradual increase in the pro-inflammatory cytokine IL-1α concentrations during the 2 weeks after SCI, with a peak reached on 14 dpi which exceeded that for the intact controls in 1.58 times (*P* < 0.05).

It was found that levels of the pro-inflammatory cytokine IL-2 in the SCI tend to decrease at all stages when compared with the same period in the intact controls. On 3, 7, and 14 dpi the level of IL-2 at the site of SCI was lower than that in the intact controls by 45.7% (*P* < 0.05), 49.4% (*P* < 0.05), and 63.6% (*P* < 0.05), respectively. The IL-5 concentration was halved (*P* < 0.05) on 3 dpi compared with the intact controls and remained at the same level for 2 weeks. A similar trend was observed for IL 18 and GM-CSF, their concentrations decreased within the first 2 weeks by 40.5–43.7% (*P* < 0.05) and 43.7–44.2% (*P* < 0.05), respectively, as compared with the intact controls.

The level of IL-17α was reduced by 6.66 times (*P* < 0. 05) on 3 dpi compared with the intact controls. On 7 and 14 dpi, the concentration of IL-17α had risen slightly, but it did not reach the baseline (intact control). On 3 and 7 dpi, the concentration of IFNγ was below that of the intact controls by ~37% (*P* < 0.05). However, on 14 dpi, the IFNγ concentrations had rebounded with no significant differences from intact controls.

During the first 2 weeks after SCI, the level of anti-inflammatory cytokine IL-10 increased in the site of injury, with the peak observed on day 7, exceeding the same parameter in the intact controls in 1.95 times (*P* < 0.05). IL-13 exhibited an opposite trend, with decreasing concentrations following SCI (*P* < 0.05).

MCP-1 concentrations initially increased sharply on day 3 after SCI (3.5-fold, *P* < 0.05) and then decreased of in the area of SCI on 7 (0.54-fold, *P* < 0.05) dpi, respectively, as compared with the intact controls. However, by day 14, the MCP-1 level returned to the baseline (intact control). There was a sharp increase of GRO KC CINC-1 on 3 dpi; it increased in 13.3 times compared with the intact controls (*P* < 0.01). On 7 and 14 dpi, its concentrations decreased, but remained 5.1 and 2.73 times higher than intact controls (*P* < 0.05).

### Blood Serum Cytokine Profile after SCI

The assessment of the cytokine profile in blood serum at different periods after SCI showed significant changes in concentration of cytokines/chemokines such as IL-1β, IL-2, MCP-1, RANTES, IL-5, MIP-1α, and IL-1α (Table 3; Figure 1).

**Table 3:** The serum concentration of cytokines at different stages after spinal cord injury.

	3 dpi	7 dpi	14 dpi	Intact control
MIP-1α	123.52 ±45.51*	1,222.38 ± 196.27**	212.03 ± 100.43*	37.2 ± 22.26
Interleukin 1 (IL-1) α	12.6 ± 0.4	49.35 ± 22*	12.3 ± 0.1	12.44 ± 0.88
IL-2	12.65 ± 1.11	18.65 ± 6.9	3.18 ± 1.66*	14.7 ± 6
IL-5	30.6 ± 8.08*	23.62 ± 6.8	6.37 ± 2.94	12.88 ± 6.82
IL-1β	28.85 ± 15*	226 ± 56.4	123.87 ± 55.4	214.64 ± 71.12
MCP-1	979.33 ± 215.79*	698.5 ± 186.04	631.12 ± 155.36	388.74 ± 247.72
RANTES	3,285.81 ± 1,550*	8,769.74 ± 2,392.76	8,195.89 ± 3,557.25	8,934.55 ± 2,106.55
IL-18	173.89 ± 104.3	328.21 ± 123.8	366.02 ± 132.72	266.6 ± 96.7
Tumor necrosis factor (TNF) α	3.4 ± 1.7	2.4 ± 0.05	2.4 ± 0.05	2.7 ± 1.3
GRO KC CINC-1	14.6 ± 0.1	23.13 ± 7.65	14.6 ± 0.1	14.85 ± 0.94
IL-12 p70	50.5 ± 30.96	26.56 ± 12.72	43.3 ± 23.6	17.49 ± 8.21
IL-10	23.66 ± 10.14	46.6 ± 20.92	18.2 ± 6.3	30.83 ± 15.07
VEGF	22.3 ± 6.83	49.71 ± 12.37	58.59 ± 22.32	31.7 ± 9.51
G-colony-stimulating factor (CSF)	2.83 ± 0.41	3.19 ± 0.79	2.43 ± 0.47	2.49 ± 0.65
GM-colony-stimulating factor (CSF)	4.9 ± 2.47	4.32 ± 0.68	4.53 ± 0.7	4.15 ± 0.36
IL-4	3.91 ± 0.68	4.06 ± 0.75	3.86 ± 0.86	3.33 ± 0.64
IFNγ	<16.5	<16.5	<16.5	<16.5
IL-6	<73.2	79.97 ± 17.92	<73.2	<73.2
IL-13	<4.9	<4.9	<4.9	<4.9
IL-17α	<2.45	<2.45	<2.45	<2.45

*P < 0.05, **P < 0.01 compared with intact control.

*<, protein concentrations below the specified value cannot be detected with the kit*.

The concentrations of pro-inflammatory cytokines such as MIP-1α and IL-1α tended to increase up to 7 dpi, by as much as ~33 times (*P* < 0.01) and ~4 times (*P* < 0.05) higher than those in the intact controls, respectively. However, by 14 dpi, the concentration of IL-1α returned to baseline and that of MIP-1α reduced to a value exceeding that in the intact controls by only 5.7 times (*P* < 0.05).

By 7 dpi, the serum inflammatory cytokine IL-2 concentration had increased in 1.27 times compared with the intact controls. However, by 14 dpi, there was an abrupt decrease of IL-2 concentrations by 78.3% (*P* < 0.05) lower than that in controls. The concentration of cytokine IL-5 increased by 3 dpi (2.37-fold, *P* < 0.05) but later, from 7 to 14 dpi, it exhibited a downward trend to control values. A sharp, by 85.6% (*P* < 0.05) drop in IL-1β levels was detected by 3 dpi, as compared with the intact controls but this had normalized at 7 and 14 dpi.

On 3 dpi, the blood serum MCP-1 level increased 2.5 times (*P* < 0.05) as compared with the intact controls. By 7–14 dpi, this had reduced to values exceeding controls in 1.62–1.8 times. A more than 0.6-fold reduction of RANTES levels (*P* < 0.05) in blood serum was detected on 3 dpi as compared with the intact controls. However, by 7 and 14 days after injury RANTES values returned to the baseline (intact control).

## Discussion

This study aimed to evaluate a unique biomarker profile in SCI. A secondary objective was to localize the inflammatory phenotype to either spinal cord or the peripheral immune system. Although inflammation is a ubiquitous response after trauma to the CNS, the overall impact of immune activation during SCI remains largely unknown. Inflammatory and autoimmune reactions aggravating degeneration processes and inhibiting neuroregeneration lead to various negative results. Therefore, it is evident that an assessment of the cytokine profile will allow for possible modulation of these posttraumatic reactions.

We have studied the changes in a systemic and local cytokine profile following SCI (acute and subacute periods) in rats compared with intact animals. The concentration of almost all studied cytokines was different in the blood serum and spinal cord tissue. We identify a tendency that most often the concentration of cytokines increases/decreases first in the spinal cord tissue and later in the blood serum. During the first 2 weeks after SCI, we detected significant changes in concentrations of 14 cytokines/chemokines with different inflammatory and immunological activities.

We found a similar upward trend of systemic and local levels of the pro-inflammatory chemokine MIP-1 α during the first 2 weeks after SCI with a maximum increase of 31 times on 3 dpi MIP-1α, being a macrophage inflammation protein, activates human granulocytes, which leads to acute neurotrophic inflammation (3, 4). Its increase in the spinal cord tissues can be associated with disruption of the blood–brain barrier and an increased migration of macrophages and neutrophils to the site of degeneration. MIP-1α also induces the synthesis and release of other pro-inflammatory cytokines such as IL-1, IL-6, and TNF-α from fibroblasts and macrophages (5). In contrast, we only detected an increased level of the pro-inflammatory cytokine IL-1α in both the injury site and peripheral blood serum, with a peak on 14 and 7 days, respectively, in rats after SCI. As well as MIP-1α, an increased IL-1α level in the site of SCI might be due to continuing migration of IL-1α expressing macrophages and neutrophils. The elevated level of this cytokine in blood increases the number of neutrophils and activates the proliferation of T-lymphocytes (6).

In our experiment, a local level of pro-inflammatory cytokine IL-2, predominantly expressed by helper T cells, decreases within 2 weeks after SCI. At the same time, blood serum IL-2 level increased to 7 dpi followed by an abrupt decline. It has been shown that the interaction of IL-2 with its own receptor leads to the proliferation of T-helpers, which then affect the differentiation and proliferation of cytotoxic T cells, NK cells, lymphokine-activated killers, B cells, and macrophages. This causes further progression of immune response (6). The increased IL-2 level on 7 dpi can be accounted for by an active immune response after SCI due to the disruption of the blood–brain barrier and the penetration of CNS antigens into the blood. It has been established that IL-2 stimulates the synthesis and secretion of a number of lymphokines such as IL-4, IL-6, IFN-γ, CSFs, and TNFs (7). Nevertheless, when examining the concentrations of IL-4, IL-6, and IFN-γ, we did not detect any quantitative and temporal changes in their levels in blood serum.

We found the opposite dynamics in changes of system and local levels of IL-5 on 3 dpi, namely, IL-5 decreased at the site of SCI and increased in blood serum. It is known that IL-5, synthesized by TH2 type lymphocytes, stimulates the growth of B cells, enhances the secretion of immunoglobulins, and is also a key activator of eosinophils (8). Its increased blood serum level on 3 dpi might indicate the activation of a humoral component of the immune system. Nevertheless, by 7 and 14 dpi, there was a reduced level of IL-5 in the site of SCI and a downward tendency in its level in blood serum.

Changes in the level of pro-inflammatory cytokine IL-1β in the form of a sharp drop in its level on 3 dpi were observed only in blood serum. Interestingly, the level of IL-1β expressed in the spinal cord tissue by microglia, astrocytes, and oligodendrocytes (5) did not undergo significant changes within 2 weeks after SCI. Maintenance of IL-1β levels in the area of SCI seems to be positive for remyelination. It was shown that IL-1β(−/−) mice failed to remyelinate properly (6). On the other hand, one of the main activities of IL-1β in the brain is the induction of reactive astrogliosis, with astrocyte activation and IL-6 production, an effect that is strongly augmented in the presence of TNF-α, IFN-γ, and an IL-6 receptor (9, 10).

We observed increased systemic and local levels of MCP-1 (CCL2) on 3 dpi. MCP-1 is involved in neuroinflammatory processes in various diseases of the CNS, which are accompanied by neuronal degeneration (4, 11). It is one of the key chemokines that regulate the migration and infiltration of monocytes and macrophages (11). MCP-1 is produced by many types of cells, including endothelial epithelial cells, fibroblasts, astrocytes, monocytes, and microglia cells (12–15). MCP-1 is likely to play an important role in the early phase of inflammation after SCI, as its value is normal in the site of SCI on 14 dpi. The results obtained are in line with data indicating the maximum expression of this chemokine between 12 h and 2 days after a brain lesion (16–19). *In situ* hybridization combined with immunohistology revealed that astrocytes are the main source of CCL2 under these conditions. In the spinal cord, CCL2 RNA upregulation was also demonstrated to be about 50-fold after a stab wound model of mechanical injury, coinciding with a microglial reaction and the accumulation of macrophages. Nevertheless, the MCP-1 level with a downward tendency remained elevated on 7–14 dpi in blood serum. It was previously demonstrated in line with our results that chemokine MCP-1 was elevated in blood serum of patients with stroke (19, 20).

On 3 dpi, we observed an enhanced concentration of GRO KC CINC-1 in the area of SCI, which still remained elevated on 7–14 dpi. GRO KC CINC-1, being a chemotactic substance for neutrophils, is expressed by macrophages, neutrophils, and epithelial cells (21). This cytokine plays a role in the development of the spinal cord by inhibiting the migration of oligodendrocyte precursors and is involved in the processes of angiogenesis, inflammation, wound healing, and oncogenesis (22). Studies in mice have shown that GRO KC CINC-1 reduces the severity of multiple sclerosis and can exhibit a neuroprotective function (23). GRO KC CINC-1 might be actively involved in the regenerative processes in the CNS during the first 2 weeks after SCI.

RANTES (CCL5) reduced levels on 3 dpi were observed only in blood serum. RANTES is chemotactic for T cells, eosinophils, and basophils. This chemokine plays an active role in recruiting leukocytes into inflammatory sites (19). In animal experiments, the increased expression of RANTES starting within 1 h after brain injury and returning to a baseline within 7 days has been described (16, 24). Unlike our study in a model of rats with a contused SCI, in a human study, there was an increase of RANTES in blood serum of patients with severe brain injury, and it tended to correlate with disease severity (25).

There was a similar tendency for IL-18 and GM-CSF levels to decrease during the first 2 weeks in the area of SCI. Both IL-18 and GM-CSF are produced by macrophages and some other types of immune cells (26). Our results for the changes in the level of pro-inflammatory cytokine IL-18 do not coincide with the data of Yatsiv et al. (27). In an adult mouse model of closed head injury, these authors demonstrated a significant increase of IL-18 levels in injured brains 7 days after trauma. Head injury to newborn rodents was also associated with increased IL-18 intracerebral synthesis. The administration of IL-18 binding protein leads to attenuated apoptotic cell death and improved neurological outcome in mice after experimental closed head injury. Hedtjärn et al. have also shown that IL-18-deficient mice had attenuated brain lesions (28). This discrepancy might be due to the small number of animals in our study as well as a marked variability of circulating IL-18 levels. GM-CSF belongs to the group of granulocyte-macrophage CSFs along with IL-3 and IL-5. The expression of GM-CSF can be inhibited by IL-10, IFNγ, and IL-4 (29). In our case, the decreased GM-CSF level seems to be associated with the activity of IL-10, whose elevated concentrations were detected at the site of SCI.

We observed reduced levels of IL-17α and IL-13 in the spine 2 weeks after injury. It has been shown that IL-17α is produced by T-helper cells, whereas IL-13 is produced predominantly by TH2 type lymphocytes (8, 30). IL-17 signaling attracts monocytes and neutrophils to the site of inflammation in response to invading pathogens (26, 31). The expression of this cytokine is also associated with autoimmune diseases (32, 33). IL-13 in turn is a cytokine of allergic inflammation (34) and promotes the proliferation of myeloid type cells (29). IL-17 and IL-13 are unlikely to be involved significantly in an early posttraumatic period after SCI.

We also detected a reduced level of IFNγ at the injury site on 3 and 7 dpi, which became normal on 14 dpi. IFNγ is known to be a potent pro-inflammatory factor that triggers the activation of microglia and the subsequent release of neurotoxic factors (35). However, our experimental model of SCI did not indicate the involvement of IFNγ in the process of posttraumatic microglia activation.

It has been shown that the activation of pro-inflammatory cytokines in the acute period of SCI is also accompanied by a simultaneous increase in the synthesis of their antagonists—anti-inflammatory IL-4 and IL-10, which promote the survival of neurons and glia in the so-called penumbra zone by inhibiting a local inflammatory response and initiating the production of nerve growth factors (4, 6). In our study, we found an elevated level of IL-10 in the site of SCI which peaked on 7 dpi. IL-10 is produced by macrophages, B cells, and Th2 cells in addition to astrocytes and microglia (36, 37). As an immunomodulator, IL-10 suppresses the activity of Th1 and NK cells. A neuroprotective effect of IL-10 was demonstrated in rats receiving IL-10 either intracranially or peritoneally during brain injury (38). An increased IL-10 level can contribute to the effective regulation of TNF-α levels by regulating the activity of an enzyme-synthesizing TNF-α (39, 40). Thus, one of the mechanisms of compensatory anti-inflammatory response syndrome seems to be involved in acute and early periods of traumatic SCI.

The cytokine imbalance that develops after SCI leads to its progression through the formation of many new “cytokine cascades” that determine the amplification and prolongation of cytokine effects and triggering of so-called “vicious circles” leading to multiple organ dysfunction/insufficiency and systemic complications (41, 42). Most often, a cytokine profile assay is carried out for demyelinating disorders (multiple sclerosis, acute disseminated encephalomyelitis, optic neuritis, and neuromyelitis optica) and other chronic autoimmune inflammatory disorders of the CNS (43). Nevertheless, an extended multiplex analysis of the cytokine profile is relevant to determine the ways of possible modulation of posttraumatic reactions in the CNS. In our study, we evaluated the cytokine profile in blood serum and the spinal cord of experimental animals with a model of dosed contusion SCI in acute and early periods after injury. Our study demonstrated that the inflammatory reaction and immunological response after SCI were not limited within the CNS but were widespread. This fact was confirmed by changes of the cytokine profile detected in blood serum samples. A further study of a complex cytokine network imbalance after SCI and determining its possible correlation with the severity of damage and clinical events seems important. This would provide a better understanding of the role cytokines play in the pathophysiology of the disease.

## Ethics Statement

All experimental procedures were performed in accordance with the Kazan (Volga Region) Federal University Animal Care and Use Committee guidelines (Permit Number: 2 dated 5 May 2015), and experimental protocols were consistent with the recommendations of the Physiological Section of the Russian National Committee on Bioethics.

## Author Contributions

YM—statistical analysis and writing an article. EA—SCI and postsurgical care. EM—multiplex analysis. SK—multiplex analysis and statistical analysis. LG—venous blood collection. AR—the development of a research plan and participation in the writing of an article.

## Conflict of Interest Statement

The authors declare that the research was conducted in the absence of any commercial or financial relationships that could be construed as a potential conflict of interest.
